# The evidence for histamine H_3_ receptor-mediated endothelium-dependent relaxation in isolated rat aorta

**DOI:** 10.1155/S0962935195000354

**Published:** 1995-05

**Authors:** D. M. Djuric, I. Z. Andjelkovic

**Affiliations:** 1 Institute of Physiology Medical Faculty University of Belgrade Visegradska 26/II P.O. Box 783 Belgrade 11000 Serbia

## Abstract

The presence of histamine H_3_ receptors was evaluated on the rat aorta endothelium. In the presence of pyrilamine (1 nM, 7 nM, 10 nM) or thioperamide (1 nM, 10 nM, 30 nM) the concentration–response curve for histamine-induced (0.1 nM − 0.01 mM) endothelium-dependent rat aorta relaxation was shifted to the right without significant change of the E_max_ indicating competitive antagonism by pyrilamine (pA_2_ = 9.33 ± 0.34, slope = 1.09 ± 0.36) or thioperamide (pA_2_ =9.31 ± 0.16, slope=0.94 ± 0.10). Cimetidine (1 μM) did not influence histamine-induced endothelium-dependent rat aorta relaxation. In the presence of thioperamide (1 nM, 10 nM, 30 nM) the concentration–response curve for (R)α-MeHA-induced (0.1 nM − 0.01 mM) endothelium-dependent relaxation was shifted to the right without significant change of E_max_ indicated competitive antagonism by thioperamide (pA_2_ = 9.21 ± 0.4, slope = 1.03 ± 0.35). Pyrilamine (100 nM) or cimetidine (1 μM) did not influence (R)α-MeHA-induced endothelium-dependent rat aorta relaxation. These results suggest the presence of a heterogenous population of histamine receptors, H_1_ and H_3_, on rat aorta endothelium.


Mediators of Inflammation 4, 217–221 (1995)

### The evidence for histamine $H_3$ receptor-mediated endothelium-dependent relaxation in isolated rat aorta

D. M. Djuric[CA] and I. Z. Andjelkovic

Institute of Physiology, Medical Faculty University of Belgrade, Visegradska 26/II, P.O. Box 783, 11000 Belgrade, Yugoslavia

[CA]Corresponding Author

**The presence of histamine $H_3$ receptors was evaluated on the rat aorta endothelium. In the presence of pyrilamine (1 nM, 7 nM, 10 nM) or thioperamide (1 nM, 10 nM, 30 nM) the concentration–response curve for histamine-induced (0.1 nM - 0.01 mM) endothelium-dependent rat aorta relaxation was shifted to the right without significant change of the $E_{max}$ indicating competitive antagonism by pyrilamine ($pA_2 = 9.33 \pm 0.34$, slope = $1.09 \pm 0.36$) or thioperamide ($pA_2 = 9.31 \pm 0.16$, slope = $0.94 \pm 0.10$). Cimetidine (1 μM) did not influence histamine-induced endothelium-dependent rat aorta relaxation. In the presence of thioperamide (1 nM, 10 nM, 30 nM) the concentration–response curve for (*R*)α-MeHA-induced (0.1 nM - 0.01 mM) endothelium-dependent relaxation was shifted to the right without significant change of $E_{max}$ indicated competitive antagonism by thioperamide ($pA_2 = 9.21 \pm 0.4$, slope = $1.03 \pm 0.35$). Pyrilamine (100 nM) or cimetidine (1 μM) did not influence (*R*)α-MeHA-induced endothelium-dependent rat aorta relaxation. These results suggest the presence of a heterogenous population of histamine receptors, $H_1$ and $H_3$, on rat aorta endothelium.**

**Key words:** Endothelium, Histamine $H_3$ receptor, Rat aorta.

### Introduction

Two types of histamine receptors, $H_1$ and $H_2$, participate in vascular responses to histamine.[1] The novel histamine $H_3$ receptors were identified as inhibitory presynaptic autoreceptors on histamine-containing nerve terminals in the rat brain cortex but have since been shown to inhibit the release of various neurotransmitters both in the central and peripheral nervous systems.[2] Recent articles provide strong evidence for the presence of histamine $H_3$ receptors at the different sites, including the rabbit middle cerebral artery endothelium,[3,4] guinea-pig aorta,[5] mesenteric artery,[6] rabbit saphenous artery,[7] guinea-pig myocardium,[8] guinea-pig ileum,[9] guinea-pig lung and bronchiole,[10,11] guinea-pig intestine,[12] porcine small intestine,[13] rabbit gastric glands,[14] human adenoidal mast cells,[15] human and rhesus monkey brain.[16]

The purpose of the present study was to determine whether histamine $H_3$ receptors are localized on rat aorta endothelium and to assess their possible role in endothelium-dependent responses.

### Materials and Methods

*Vascular preparations:* Male Wistar rats weighing between 100 and 200 g were stunned and the thoracic aorta was excised and dissected free of surrounding tissue. Ring segments (4 mm) were prepared and fixed isometrically in 20 ml organ bath containing Tyrode's solution of the following composition (mM): NaCl 136.9, KCl 2.69, $CaCl_2$ 1.8, $MgCl_2$ 1.05, $NaHCO_3$ 11.9, $NaH_2PO_4$ 0.42 and glucose 5.55 at 37°C under a moderate tension of 1 g for 90 min (the optimal point of its length-tension curve as determined from the tension developed in response to potassium chloride 40 mM) and gassed with 95% $O_2$/5% $CO_2$. The preparations were precontracted by phenylephrine (300 nM). In some preparations the endothelium was removed mechanically by gentle and careful rubbing of the intimal surface with a stainless steel wire (31 gauge diameter) in order to avoid stretching and damaging of the vascular smooth muscle cells. The presence of endothelium was confirmed by using acetylcholine (300 nM). The failure of acetylcholine to induce relaxation of preparations was taken as an indication of endothelium removal.

*Experimental procedure:* After the equilibration period, concentration–response curves were obtained by cumulative addition of histamine (0.1 nM – 0.01 mM) or (*R*)α-methylhistamine ((*R*)α-MeHA, 0.1 nM – 0.01 mM) on precontracted preparations alone or in the presence of pyrilamine (1 nM, 7 nM, 10 nM for histamine and

© 1995 Rapid Communications of Oxford Ltd

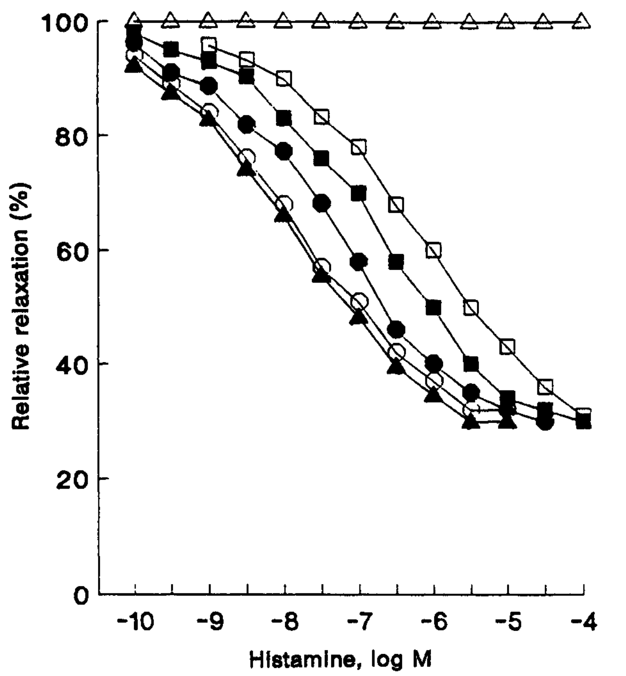

FIG. 1. Concentration-response curves for histamine in rat aorta with intact endothelium alone or in the presence of pyrilamine or cimetidine. The data are expressed as means ($n=6$). (Values for S.E.M. are excluded from the figure). Histamine (▲); pyrilamine, 1 nM (●); pyrilamine, 7 nM (■); pyrilamine, 10 nM (□); cimetidine, 1 μM (○); denuded endothelium (△).

100 nM for (*R*)α-MeHA), cimetidine (1 μM for both histamine and (*R*)α-MeHA) or thioperamide (1 nM, 10 nM, 30 nM for both histamine and (*R*)α-MeHA).

All drugs were added directly to the bath in a volume of 150 μl and the concentrations given are the calculated final concentrations in the bath solution. When potassium chloride was used as a spasmogen, the stated concentration excluded the potassium chloride already present in Tyrode's solution.

*Data analysis:* Responses are expressed as a percentage of the maximal relaxation induced by papaverine (100%, 0.1 mM). The slopes of the log concentration–response curves, correlation coefficients ($r$), $E_{max}$ (maximum response) and $pA_2$ (−log molar concentration of antagonist reducing the agonist response by a factor of 2) values were evaluated from concentration–response curves plotted for each agonist in the presence of different antagonists. For calculating these different values the data are expressed as means ± S.E.M; $n$ refers to the number of experiments. $E_{max}$ values were compared using Student's *t*-test. $p$ values less than 0.05 were considered to be significant.

*Drugs:* The following compounds were used: acetylcholine chloride (Sigma), phenylephrine hydrochloride (Sigma), histamine dihydrochloride (Sigma), (*R*)α-methylhistamine (Research Biochemicals Incorporated), pyrilamine maleate (Sigma), cimetidine (Sigma) and thioperamide maleate (Research Biochemicals Incorporated). All solutions were prepared immediately before the experiment and stored on ice until use, except thioperamide which was dissolved in dimethylsulfoxide. (Previous experiments had shown that the solvents used had no effects on the preparations).

### Results

*Responses to histamine:* Histamine (0.1 nM – 0.01 mM) induced a concentration-dependent relaxation of rat aorta precontracted with phenylephrine (300 nM), with intact endothelium reaching approximately 70% of the papaverine-induced maximum relaxation (0.1 mM). Removal of the endothelium abolished the relaxation to histamine.

When pyrilamine, a potent and selective histamine $H_1$ antagonist ($K_d$ for $H_1$ = 0.8 nM, $K_d$ for $H_2$ = 5.2 μM, $K_d$ for $H_3$ = > 3 μM, see Reference 2) was present (1 nM, 7 nM, 10 nM), the concentration–response curve for histamine-induced rat aorta relaxation was shifted to the right without significant change of the $E_{max}$. Schild plot analysis indicated that antagonism by this compound was competitive. The slope for the regression curve was 1.09 ± 0.36 with a $pA_2$ value of 9.33 ± 0.34 ($r = 0.949$) (Fig. 1).

The potent and selective $H_2$ antagonist, cimetidine (1 μM, $K_d$ for $H_2$ = 0.8 μM, $K_d$ for $H_1$ = 0.45 mM, $K_d$ for $H_3$ = 33 μM, see Reference 2) did not influence histamine-induced endothelium-dependent rat aorta relaxation (Fig. 1).

When thioperamide, a potent and selective histamine $H_3$ antagonist ($K_d$ for $H_1$ > 100 μM, $K_d$ for $H_2$ > 10 μM, $K_d$ for $H_3$ = 4.3 nM, see Reference 20) was present (1 nM, 10 nM, 30 nM), the concentration–response curve for histamine-induced relaxation was also shifted to the right without significant change of the $E_{max}$. Schild plot analysis indicated that antagonism by this compound was competitive. The slope for the regression curve was 0.94 ± 0.10 with a $pA_2$ value of 9.31 ± 0.16 ($r = 0.993$) (Fig. 2).

*Responses to (R) α-MeHA:* The potent and selective $H_3$ agonist, (*R*)α-MeHA (0.1 nM – 0.01 mM) induced a concentration-dependent relaxation of rat aorta precontracted with phenylephrine (300 nM), with intact endothelium reaching

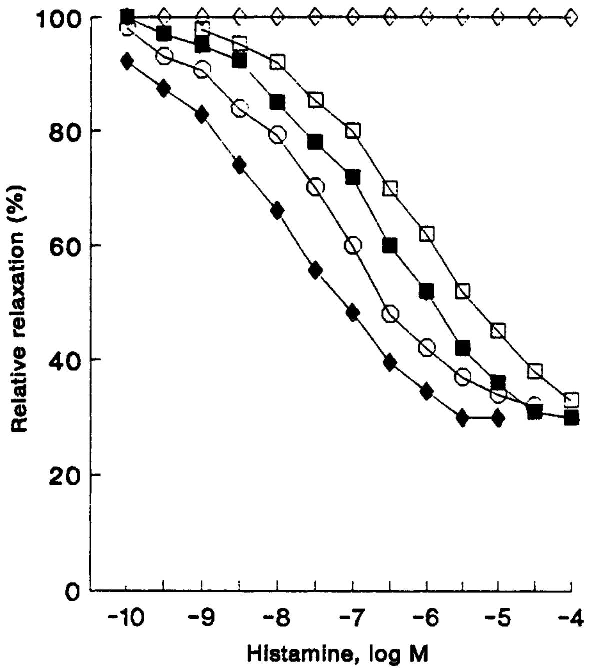

FIG. 2. Concentration-response curves for histamine in rat aorta with intact endothelium alone or in the presence of thioperamide. The data are expressed as means ($n = 6$). (Values for S.E.M. are excluded from the figure). Histamine (◆); thioperamide, 1 nM (○); thioperamide, 10 nM (■); thioperamide, 30 nM (□); denuded endothelium (◇).

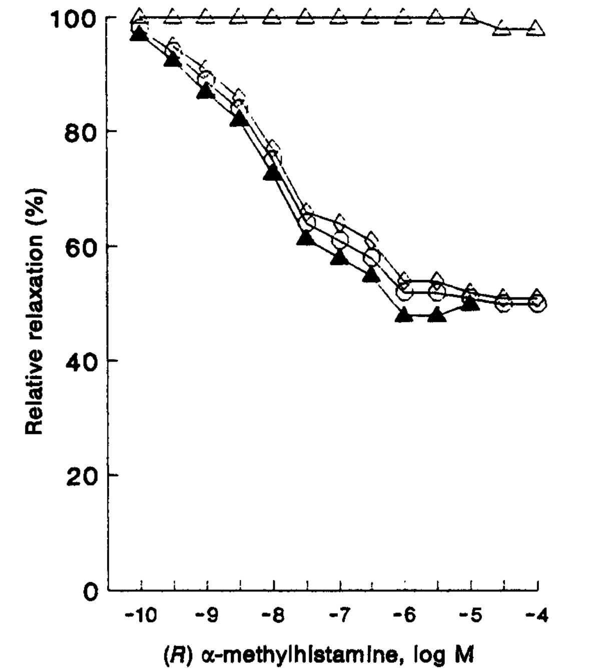

FIG. 3. Concentration-response curves for (*R*)α-MeHA in rat aorta with intact endothelium alone or in the presence of thioperamide. The data are expressed as means ($n = 6$). (Values for S.E.M. are exluded from the figure). (*R*)-α-methylhistamine (▲); pyrilamine, 100 nM (○); cimetidine, 1 μM (◇); denuded endothelium (△).

approximately 50% of the papaverine-induced maximum relaxation (0.1 mM). Removal of the endothelium abolished the relaxation to (*R*)α-MeHA.

Pyrilamine (100 nM) or cimetidine (1 μM) did not influence (*R*)α-MeHA-induced endothelium-dependent rat aorta relaxation (Fig. 3).

When thioperamide (10 nM, 30 nM, 100 nM) was present, the concentration–response curve for (*R*)α-MeHA-induced relaxation was shifted to the right without a significant change of the $E_{max}$. Schild plot analysis indicated that antagonism by this compound was competitive. The slope for the regression curve was $1.03 \pm 0.35$, with a $pA_2$ value of $9.21 \pm 0.40$ (Fig. 4).

### Discussion

Histamine is present in essentially all tissues and it can stimulate all three classes of histamine receptors. It is found in significant concentrations in the blood and also in the vessel walls.[17] Intravascular administration of histamine elicits a concentration-dependent fall in blood pressure in most species. Many studies have indicated the involvement of $H_1$ and $H_2$ receptors in this depressor response.[18]

The histamine $H_3$ receptors were found within the central nervous system of the rat and the human where they appear to be involved in the feedback control of both histamine synthesis and release at the level of histaminergic nerve endings.[19,20] Furthermore, stimulation of histamine $H_3$ receptors has been shown to inhibit adrenergic and cholinergic neurotransmission in the peripheral autonomic nervous system.[6,12] There is some controversy about whether histamine $H_3$ receptors are present on the sympathetic nerve fibres innervating blood vessels.[6] In two isolated vessels, the rabbit middle cerebral artery[3,4] and guinea-pig aorta,[5] a potent and selective histamine $H_3$ agonist, (*R*)α-MeHA produced relaxation probably via stimulation of postsynaptic histamine $H_3$ receptors. These findings suggest that depending on the species and the experimental model, several mechanisms (activation of pre- and postsynaptic histamine $H_3$ receptors or histamine $H_3$ receptor-independent mechanisms) contribute to the overall effect of (*R*)α-MeH on cardiovascular function.[22] Experiments with the Langendorff perfusion of the guinea-pig heart have shown that the histamine $H_3$ agonist *N*-α-methylhistamine (*N*-α-MeH) produces an increase in coronary flow and a decrease in coronary vascular resistance which

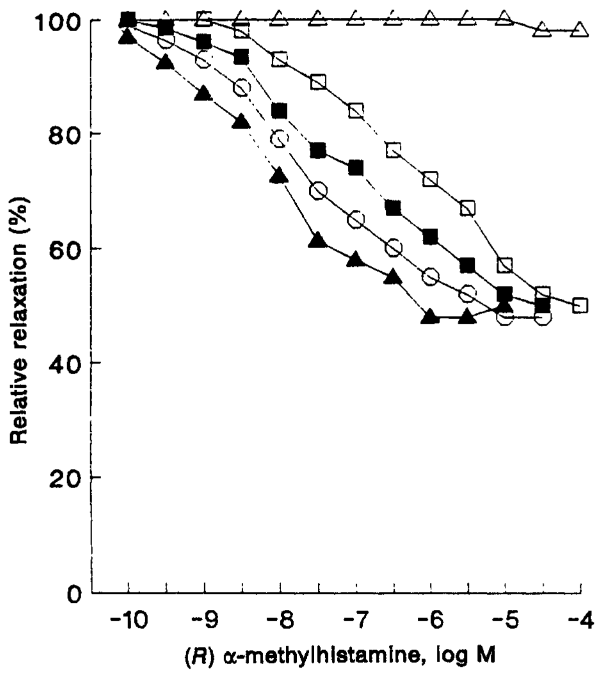

FIG. 4. Concentration–response curves for (*R*)α-MeHA in rat aorta with intact endothelium, alone or in the presence of pyrilamine or cimetidine. The data are expressed as means ($n = 6$). (Values for S.E.M. are excluded from the figure). (*R*)-α-methylhistamine (▲); thioperamide, 1 nM (○); thioperamide, 10 nM, (■); thioperamide, 30 nM (□); denuded endothelium (△).

could be abolished by a mixture of impromidine (a non-selective histamine $H_3$ antagonist with $H_2$ agonistic effects) and cimetidine.[23]

The role of the endothelium in the relaxation of precontracted blood vessel preparations has been demonstrated for different physiologically important substances. The study presented here showed that the presence of endothelium is also essential for the relaxing effect of histamine and (*R*) α-MeHA on the isolated rat aorta. The physiological role of the endothelium in histamine-induced relaxation could be of physiological importance. Sensitivity to histamine could also be higher in blood vessels with a more important role in the regulation of peripheral resistance.

The results with both histamine and pyrilamine suggest the presence of histamine $H_1$ receptors on rat aorta endothelium which is in agreement with results of the other authors.[24–26] Thioperamide antagonizes both histamine and (*R*)α-MeHA-induced relaxations, resulting in about the same $pA_2$ values (9.31 for histamine and 9.21 for (*R*)α-MeHA, respectively). These $pA_2$ values are close to that (8.96) found for blockade of histamine $H_3$-mediated inhibition of [$^3$H]-histamine release in rat brain slices.[20] The $pA_2$ value of the histamine $H_3$ antagonist thioperamide was very similar to its values for various responses mediated by histamine $H_3$ receptors.[27] Schild plots for histamine (agonist of all three classes of histamine receptors) remain straight both in the presence of pyrilamine or thioperamide. This is not surprising, although the histamine vascular effects in different biological species involve two receptor systems, the histamine $H_1$ and histamine $H_2$ receptors. New observations suggest that histamine $H_3$ receptors are also localized at the postsynaptic level in the rabbit middle cerebral artery endothelium,[3,4] guinea-pig aorta[5] and in the epithelial wall of guinea-pig bronchioles[10] and that they act on the smooth muscle.

In conclusion, there is a heterogenous population of histamine receptors, $H_1$ and $H_3$, on rat aorta endothelium which could participate in endothelium-dependent responses to histamine and its derivatives. The effects we observed might also involve NO release, cGMP accumulation and $K^+$ ions (our unpublished observations). Such events already have been implicated in histamine $H_3$ receptor-mediated endothelium-dependent relaxation in the rabbit middle cerebral artery.[4]

### References

1. Konishi M, Toda N, Yamamoto M. Different mechanisms of action of histamine in isolated arteries of the dog. *Br J Pharmacol* 1981; **74:** 111–118.
2. Hill JS. Distribution, properties and functional characteristics of three classes of histamine receptor. *Pharmacol Rev* 1990; **42:** 45–83.
3. Ea-Kim L, Oudart N. A highly potent and selective $H_3$ agonist relaxes rabbit middle cerebral artery, *in vitro*. *Eur J Pharmacol* 1988; **150:** 393–396.
4. Ea-Kim L, Javellaud J, Oudart N. Endothelium-dependent relaxation of rabbit middle cerebral artery to a histamine $H_3$-agonist is reduced by inhibitors of nitric oxide and prostacyclin synthesis. *Br J Pharmacol* 1992; **105:** 103–106.
5. Rosic M, Collis SC, Andjelkovic I, Segal M, Djuric D, Zlokovic B. The effects of (*R*)alpha-methylhistamine on the isolated guinea pig aorta. In: Timmerman H, Van der Goot H, eds. *New Perspectives in Histamine Research*. Basel, Boston, Berlin: Birkhauser Verlag, 1991: 283–287.
6. Ishikawa S, Sperelakis N. A novel class ($H_3$) of histamine receptors on perivascular nerve terminals. *Nature* 1987; **327:** 158–160.
7. Oike M, Kitamura K, Kuriyama H. Histamine $H_3$-receptor activation augments voltage-dependent $Ca^{2+}$ current via GTP hydrolysis in rabbit saphenous artery. *J Physiol (London)* 1992; **448:** 133–152.
8. Luo XX, Tan YH, Sheng BH. Histamine $H_3$-receptors inhibit sympathetic neurotransmission in guinea pig myocardium. *Eur J Pharmacol* 1991; **204(3):** 311–314.
9. Hew RWS, Hodgkinson SR, Hill SJ. Characterization of histamine $H_3$-receptors in guinea pig ileum with $H_3$-selective ligands. *Br J Pharmacol* 1990; **101:** 621–624.
10. Burgaud JL, Javellaud J, Oudart N. Bronchodilator action of an agonist for histamine $H_3$-receptors in guinea pig perfused bronchioles and lung parenchymal strips. *Lung* 1992; **170(2):** 95–108.
11. Burgaud JL, Oudart N. Bronchodilatation of guinea-pig perfused bronchioles induced by the $H_3$-receptor for histamine: role of epithelium. *Br J Pharmacol* 1993; **109:** 960–966.
12. Coruzzi G, Poli E, Bertaccini G. Histamine receptors in isolated guinea pig duodenal muscle: $H_3$ receptors inhibit cholinergic neurotransmission. *J Pharmacol Exp Ther* 1991; **258(1):** 325–331.
13. Schworer H, Katsoulis S, Racke K. Histamine inhibits 5-hydroxytryptamine release from porcine small intestine: involvement of $H_3$ receptors. *Gastroenterology* 1992; **102(6):** 1906–1912.
14. Bado A, Moizo L, Laigneau JP, Lewin MJ. Pharmacological characterization of histamine $H_3$ receptors in isolated rabbit gastric glands. *Am J Physiol* 1992; **262(1 Pt 1):** G56–61.
15. Bent S, Fehling U, Braam U, Schunack W, Schmutzler W. The influence of $H_1$-, $H_2$- and $H_3$-receptors on the spontaneous and ConA induced histamine release from human adenoidal mast cells. *Agents Actions* 1991; **33(1–2):** 67–70.

16. Martinez-Mir MI, Pollard H, Moreau J, *et al.* Three histamine receptors ($H_1$, $H_2$ and $H_3$) visualized in the brain of human and non-human primates. *Brain Res* 1990; **526(2):** 322–327.
17. Ryan MS, Brody MS. Neurogenic and vascular stores of histamine in the dog. *J Pharmacol Exp Ther* 1972; **181:** 183–186.
18. Owen DAA. Histamine receptors in the cardiovascular system. *Gen Pharmacol* 1977; **8:** 141–144.
19. Arrang JM, Garbarg M, Schwartz JC. Auto-inhibition of brain histamine release mediated by a novel class ($H_3$) of histamine receptor. *Nature* 1983; **302:** 832–837.
20. Arrang JM, Garbarg M, Lancelot JC, *et al.* Highly potent and selective ligands for histamine $H_3$-receptors. *Nature* 1987; **327:** 117–123.
21. Ichinose M, Stretton CD, Schwartz JC, Barnes PJ. Histamine $H_3$-receptors inhibit cholinergic neurotransmission in guinea-pig airways. *Br J Pharmacol* 1989; **97:** 13–16.
22. Malinowska B, Schlicker E. $H_3$ receptor-mediated inhibition of the neurogenic vasopressor response in pithed rats. *Eur J Pharmacol* 1991; **205:** 307–310.
23. Andjelkovic ZI, Collis SC, Rosic MA, Segal BM, Zlokovic ZB. Effects of Nα-methylhistamine on the isolated guinea pig heart, evidence for $H_3$-histamine receptors. *J Physiol* 1990; **424:** 58.
24. Van de Voorde J, Leusen I. Role of the endothelium in the vasodilator response of rat thoracic aorta to histamine. *Eur J Pharmacol* 1983; **87:** 113–120.
25. Van de Voorde J, Leusen I. Effect of histamine on aorta preparations of different species. *Arch Int Pharmacodyn* 1984; **268:** 95–105.
26. Carrier OG, White ER, Kirby LM. Histamine-induced relaxation of rat aorta. *Blood Vessels* 1984; **21:** 180–183.
27. Arrang JM, Devaux B, Chodkiewicz JP, Schwartz JC. $H_3$-receptors control histamine release in human brain. *J Neurochem* 1988; **51:** 105–108.

**Received 3 January 1995; accepted in revised form 21 March 1995**
